# Diagnostic value of combined pleural interleukin-33, adenosine deaminase and peripheral blood tuberculosis T cell spot detection TB for tuberculous pleurisy

**DOI:** 10.1186/s12879-021-06575-w

**Published:** 2021-08-23

**Authors:** Jin Fenhua, Wang Daohui, Lin Hui, Xia Xiaodong, Huang Wen

**Affiliations:** 1 Department of Respiratory Medicine, The Second Affiliated Hospital of Wenzhou Medical University, 109, Xueyuan Western Road, Wenzhou, 325027 Zhejiang People’s Republic of China; 2 Department of Nephrology Medicine, The Second Affiliated Hospital of Wenzhou Medical University, 109, Xueyuan Western Road, Wenzhou, 325027 Zhejiang People’s Republic of China

**Keywords:** Tuberculosis, Pleural effusion, Interleukin-33, Adenosine deaminase, Tuberculosis T cell spot detection

## Abstract

**Background:**

To investigate the correlation between pleural fluid interleukin-33 (IL-33) and adenosine deaminase (ADA) and peripheral blood tuberculosis T cell spot detection (T-SPOT.TB), and the combined value of the three tests for the diagnosis of tuberculous pleurisy.

**Methods:**

79 patients with pleural effusion admitted from June 2017 to December 2018 were enrolled. They were divided into tuberculous pleural effusion (TPE) group (57 cases, 72.2%) and malignant pleural effusion group (17 cases, 21.5%), pneumonia-like pleural effusion group (5 cases, 6.3%). Correlation between pleural fluid IL-33, pleural effusion ADA and peripheral blood T-SPOT.TB was analyzed, comparison of the three separate and combined diagnostic efficacy was also performed.

**Results:**

The levels of IL-33, ADA and peripheral blood T-SPOT.TB in patients with TPE were significantly higher than those in non-TPE (P < 0.001). The level of pleural fluid IL-33 was positively correlated with pleural effusion ADA and peripheral blood T-SPOT.TB. The Area under the ROC curve (AUC) of TPE diagnosed by pleural IL-33, ADA and peripheral blood T-SPOT.TB were 0.753, 0.912 and 0.865, respectively. AUC for combined detection of pleural effusion IL-33, ADA and peripheral blood T-SPOT.TB is the largest, with a value of 0.962. Specificity is 100% and sensitivity is 88.5%.

**Conclusion:**

Combined detection of pleural effusion IL-33, ADA and peripheral blood T-SPOT.TB can improve the diagnostic efficacy of tuberculous pleurisy.

**Supplementary Information:**

The online version contains supplementary material available at 10.1186/s12879-021-06575-w.

## Background

Tuberculous pleurisy is a common form of extrapulmonary tuberculosis. Pleural biopsy and bacteriological testing are the gold standard for diagnosis of tuberculous pleurisy. However, it is difficult to diagnose because of the invasive procedure of pleural biopsy and the difficulty in cultivating mycobacterium tuberculosis [1, 2]. With the advances in enzymology and molecular biology in recent years, enzymes and cytokines have attracted more and more attention in the pathogenesis of various immune diseases [3]. Adenosine deaminase (ADA) is present in various tissues of human body and is mainly involved in the decomposition of purine nucleosides. It has been widely used in clinical diagnosis of tuberculosis in recent years [4]. ADA testing is currently recognized as an ideal indicator for the diagnosis of tuberculous pleurisy [5]. However, several clinical conditions may increase pleural ADA levels such as parapneumonic effusions, lymphomas, solid tumors, connective tissue diseases and other infectious diseases. Therefore, new biomarkers are needed. Tuberculosis T cell spot detection (T-SPOT.TB) is a kind of interferon-γ release assay (IGRA). The number of T cells secreting interferon-γ (IFN-γ) can be used as an auxiliary method for early diagnosis of tuberculosis [6, 7]. Researches [8–10] showed that the level of interleukin-33 (IL-33) in pleural fluid was significantly higher in patients with tuberculous pleurisy than in other causes. Therefore, we speculate that IL-33 may play an important role in the production of tuberculous pleural effusion (TPE). We believe a combination of diagnostic biomarkers are useful in specific situations. The combined value of the three tests for the diagnosis of tuberculous pleurisy was investigated to identify the correlation between pleural fluid interleukin-33 (IL-33), adenosine deaminase (ADA)and peripheral blood tuberculosis T cell spot detection (T-SPOT.TB). It is intended to provide a reference for the clinical diagnosis of tuberculous pleurisy.

## Methods

### Research object

This was a cross sectional observational study conducted over 79 patients with pleural effusions at Department of Respiratory Medicine, Second Affiliated Hospital of Wenzhou Medical University from June 2017 to February 2018. The patients were divided according to pleural fluid diagnosis results into; Group of TPE (57 cases, 72.2%), and Group of non-TPE which is subdivide into; malignant pleural effusion group (17 cases, 21.5%), and pneumonia-like pleural effusion group (5 cases, 6.3%) (Additional file 1).

### Diagnostic criteria

#### Group of TPE

① Acid-fast bacilli detected in pulmonary effusion, and/or granulomatous changes in pleural biopsy samples, exclude other causes of granulomatous pleurisy;
② Exudate, pleural effusion absorption and clinical symptoms relieved by anti-tuberculosis treatment.

Diagnosis of TPE can be made if any one of them is satisfied. Suspected TB patients were with anti-tuberculosis drugs during hospitalization. Those patients whose symptoms were relieved by anti-tuberculosis treatment during hospitalization were also included in this study.

#### Group of non-tuberculous pleural effusion (non-TPE)

Including malignant pleural effusion group and pneumonia-like pleural effusion group.

Malignant pleural effusion (MPE) group: Metastatic tumor cells detected with Pleural effusion exfoliative cytology.

Pneumonia-like pleural effusion (PPE) group: presence of symptoms of cough, and fever; lung exudation showed by chest imaging and absorption of pleural fluid after antibiotic treatment.

### Exclusion criteria

Any one of the followings: ① Patients had chest trauma or received any treatment for invasive pleural examination in the previous year before hospitalization; ② Patients have received any anti-tumor or anti-tuberculosis treatment before; ③ Patients who have used glucocorticoids, non-steroidal anti-inflammatory drugs, or immunosuppressants; ④ Unknown causes of pleural effusion; ⑤ pleural effusion caused by rheumatic immune diseases.

### Specimen collection method

5 mL of drainage fluid was collected by ultrasound-guided thoracentesis from patients with pleural effusion, and centrifuged for 10 min at 3000 r/min using heparin at concentration of 500 U/ml. As per the manufacturer’s instructions, the supernatant was collected and stored in a refrigerator at 80 °C below zero for less than 1 year.

### Detection method

The IL-33 in pleural effusion was measured by enzyme linked immunosorbent assay (ELISA). The kit was provided by Abcam company (UK). The procedure was carried out strictly in accordance with the operating instructions. The levels of pleural ADA and serum lactate dehydrogenase (LDH), peripheral blood T-SPOT.TB were measured in the Second Affiliated Hospital of Wenzhou Medical University in accordance with the instructions.

### Statistical analysis

All statistical analyses were performed using Statistical Package for the Social Sciences (version 22.0; IBM Corp., Armonk, NY, USA). It was expressed as mean ± standard deviation (x ± s) for normal distributed date. Non-normally distributed data was expressed as median and interquartile range (IQR). Measurement data were compared using the independent sample T test. Pearson correlation was employed to analysis the correlations between pleural fluid IL-33, pleural effusion ADA and peripheral blood T-SPOT. The receiver operating characteristic curve (ROC curve) was plotted with the sensitivity as the Y-axis and 1-specificity as the x-axis. The optimal threshold was determined according to the Yoden index (sensitivity + specificity − 1). P < 0.05 was taken as a statistically significant difference.

### Ethics statement

This study was approved by the institutional review board of the second affiliated hospital of WenZhou medical university (Approval No L-2020-1). Written informed consent was provided by all patients.

## Results

### Demographic characteristics

A total of 79 patients were included; 57 patients had TPE, their ages were 38 ± 18 years and 72% were male. 17 patients had MPE, their ages were 64 ± 14 years and 47% were male. 5 patients had PPE, their ages were 60 ± 11 years and 60% were male. No significant difference for the distribution of age and sex among those three groups (Table 1).

**Table 1:** Demographic characteristics of patients with pleural effusion

Diagnose	N	Age (years, x ± s)	Male (n, %)	P value
TPE	57	38 ± 18	41, 72%	
MPE	17	64 ± 14	8, 47%	
PPE	5	60 ± 11	3, 60%	0.24

No significant difference for age and sex among those three groups

### Levels of IL-33, ADA, LDH and T-SPOT.TB in peripheral blood

The concentration of IL-33 in the TPE group (144.60 ± 48.10 ng/ml) was significantly higher compared to the non-TPE group (99.77 ± 35.18 ng/ml; P < 0.01, n = 79). Meanwhile, the levels of ADA in TPE (48.7 ± 14.0 U/l) were also significantly higher than non-TPE (20.8 ± 19.5 U/l, P < 0.001). Peripheral blood T-SPOT.TB levels were much higher in TPE group (171.0 ± 121.2 pg/ml) compared to non-TPE group (34.2 ± 47.6 pg/ml, P < 0.001). There were no difference in the distribution of LHD between TPE group and non-TPE group (Table 2).

**Table 2:** Levels of IL-33, ADA, LDH and peripheral blood T-SPOT.TB in pleural effusions of patients

	TPE	Non-TPE (x ± s)	P value
ADA (U/l)	48.7 ± 14.0	20.8 ± 19.5	< 0.001
LDH (U/l)	417.0 ± 188.7	562.4 ± 446.6	0.145
IL-33 (ng/l)	144.60 ± 48.10	99.77 ± 35.18	< 0.001
T-SPOT.TB (pg/ml)	171.0 ± 121.2	34.2 ± 47.6	< 0.001

The levels of pleural fluid IL-33, ADA and peripheral blood T-SPOT.TB were higher in patients with TPE (P < 0.05)

### Correlations

**Fig. 1:**
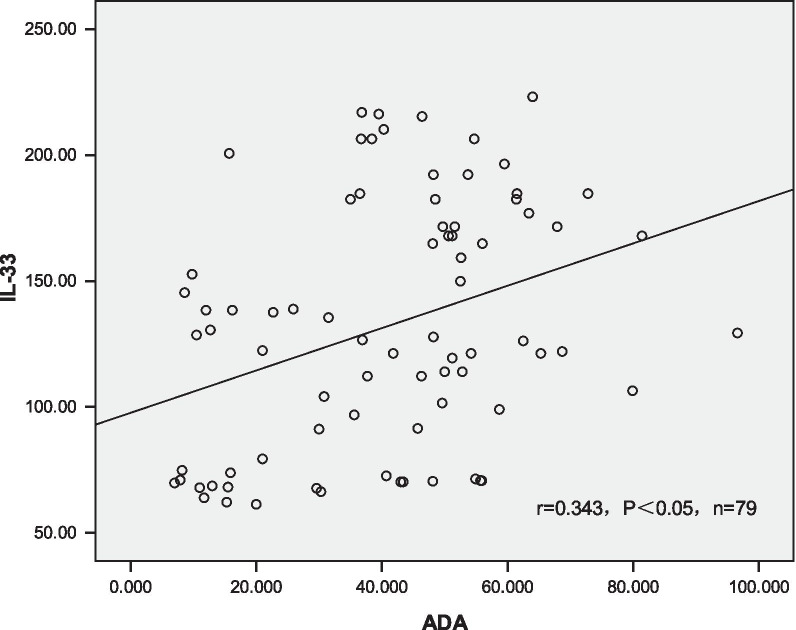
Entitled correlation between pleural fluid IL-33 level and pleural ADA level. All patients were included (n = 79). We used Pearson correlation to analysis the correlations between pleural fluid IL-33, pleural effusion ADA and peripheral blood T-SPOT. The result show that pleural fluid IL-33 level and pleural ADA level were positively linear related, (r = 0.343, P < 0.05)

**Fig. 2:**
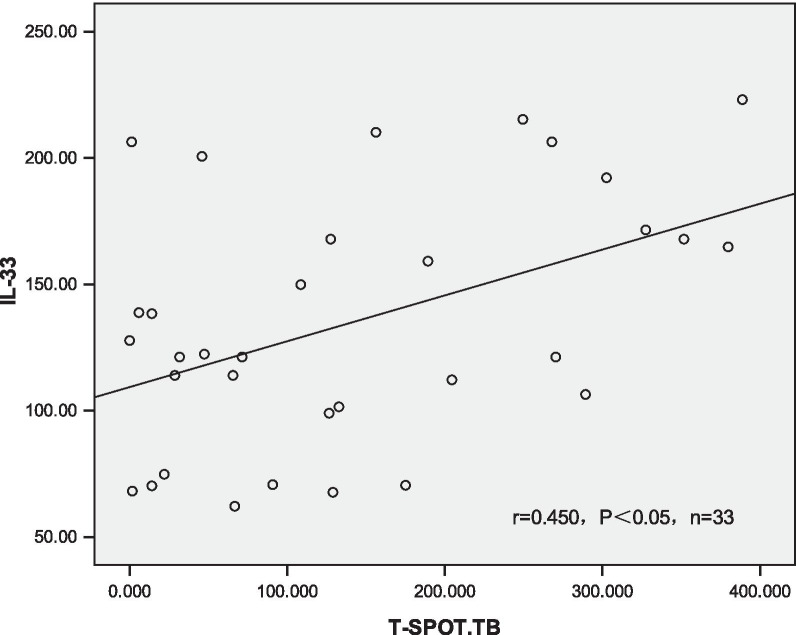
Entitled correlation between IL-33 level in pleural fluid and peripheral blood T-SPOT.TB level. 33 patients were tested for peripheral blood T-SPOT (n = 33). TB and included. Pearson correlation was employed to analysis the correlations between pleural fluid IL-33, pleural effusion ADA and peripheral blood T-SPOT. The result show that pleural fluid IL-33 level and peripheral blood T-SPOT.TB were positively related, (r = 0.450, P < 0.05)

The level of IL-33 in pleural fluid was both positively linear correlated with pleural effusion ADA and peripheral blood T-SPOT.TB (r = 0.343, 0.450, P < 0.05 respectively, shown in Figs. 1, 2).

### Diagnostic value of ADA, IL-33 and peripheral blood T-SPOT.TB for tuberculous pleurisy in pleural effusion

**Fig. 3:**
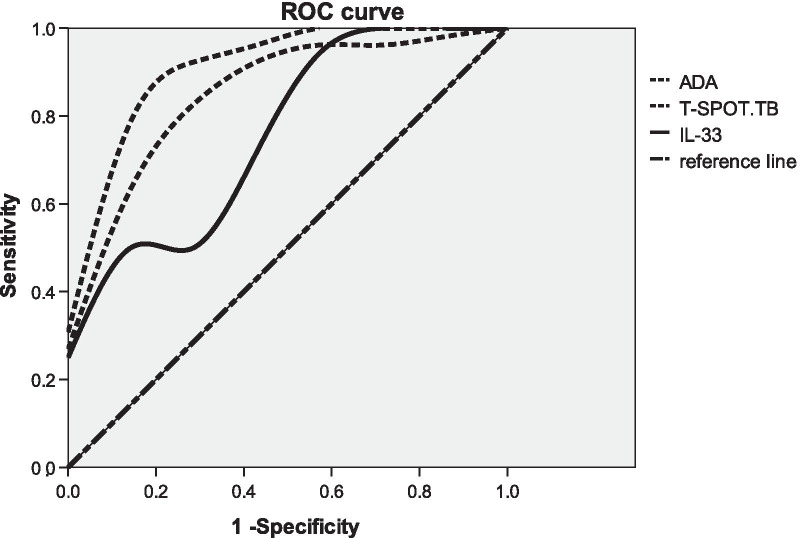
ROC curve of pleural effusion ADA, IL-33 and peripheral blood T-SPOT.TB. The receiver operating characteristic curve (ROC curve) was plotted with the sensitivity as the Y-axis and 1-specificity as the x-axis. AUC were 0.753, 0.912, 0.865 respectively for pleural IL-33, pleural ADA and peripheral blood T-SPOT (n = 79, 79, 33 for pleural IL-33, pleural ADA and peripheral blood T-SPOT respectively)

The area under curve (AUC) of IL-33 ADA peripheral blood T-SPOT.TB to differentiate TPE from all non-TB effusions AUC were 0.753 (95% CI 0.637–0.869), 0.912 (95% CI 0.804–1.000), 0.865 (95% CI 0.713–1.000) respectively for pleural IL-33, pleural ADA and peripheral blood T-SPOT (n = 79, 79, 33 for pleural IL-33, pleural ADA and peripheral blood T-SPOT respectively). The sensitivities were 49.1%, 93%, 92.3% with specificity of 100%, 90.9%, 71.4% respectively using the cut-off value of 155.96 ng/l for IL-33, 30.55 U/l for ADA, 25.35 pg/ml for T-SPOT.TB respectively (Fig. 3 and Table 3).

**Table 3:** Diagnostic value of IL-33, ADA and peripheral blood T-SPOT.TB in pleural effusion for tuberculous pleurisy

	Cut-off value	Sensitivity (%)	Specificity (%)	AUC (95% CI)
IL-33	155.96 ng/l	49.1	100.0	0.753 (0.637–0.869)
ADA	30.55 U/l	93.0	90.9	0.912 (0.804–1.000)
T-SPOT.TB	25.35 pg/ml	92.3	71.4	0.865 (0.713–1.000)

AUC were 0.753, 0.912, 0.865 respectively for pleural IL-33, pleural ADA and peripheral blood T-SPOT. The sensitivities were 49.1%, 93%, 92.3% with specificity of 100%, 90.9%, 71.4% respectively using the cut-off value of 155.96 ng/l for IL-33, 30.55 U/l for ADA, 25.35 pg/ml for T-SPOT.TB respectively

### Diagnostic value of combined detection of pleural effusion IL-33, ADA and peripheral blood T-SPOT.TB for tuberculous pleurisy

Combined IL-33 and ADA increased the diagnostic sensitivity to 92.3% for discriminating TPE from all non-TBE at the cost of specificity (Table 4). Combined IL-33 and T-SPOT.TB. decreased the diagnostic sensitivity to 76.9% and increased the specificity to 100%. The sensitivity and specificity for combined test of ADA and T-SPOT.TB were 84.6%, 100% respectively. When combined IL-33, ADA and T-SPOT.TB, AUC was the largest with sensitivity of 88%, specificity of 100% (Table 4). Both sensitivity and specificity were higher compared to the separate test for TPE and non-TPE, indicating that the combined test had a better diagnostic value than the use of a single index.

**Table 4:** Diagnostic value of combined detection of pleural effusion IL-33, ADA and peripheral blood T-SPOT.TB for tuberculous pleurisy

Combined detection	AUC	Sensitivity (%)	Specificity (%)
IL-33 + ADA	0.940	92.3	85.7
IL-33 + T-SPOT.TB	0.923	76.9	100
ADA + T-SPOT.TB	0.945	84.6	100
IL-33 + ADA + T-SPOT.TB	0.962	88.5	100

Diagnostic value of combined detection of pleural effusion IL-33, ADA and peripheral blood T-SPOT.TB for tuberculous pleurisy. Combined IL-33, ADA, TSPOT.TB, the diagnostic specificity was 100%, and the sensitivity was 88.5%

The positive predictive value and negative value for IL-33, ADA, T-SPOT.TB were 100.0%, 43.1%, 96.4%, and 83.3%, 89.8%, 80.0% respectively. The positive predictive value for combined pleural IL-33, ADA and blood T-SPOT.TB was 100.0%, and negative predictive value was 75.9%, which was the highest (Table 5).

**Table 5:** The positive predictive value and negative predictive value of the indices, alone and in combinations

Test method			Gold standard	
			Positive	Negative
IL-33	PPV 100.0%	Positive	28	0
	NPV 43.1%	Negative	29	22
ADA	PPV 96.4%	Positive	53	2
	NPV 83.3%	Negative	4	20
T-SPOT.TB	PPV 89.8%	Positive	53	6
	NPV 80.0%	Negative	4	16
IL-33 + ADA	PPV 94.6%	Positive	53	3
	NPV 82.6%	Negative	4	19
IL-33 + T-SPOT.TB	PPV 100.0%	Positive	44	0
	NPV 62.9%	Negative	13	22
ADA + T-SPOT.TB	PPV 100%	Positive	48	0
	NPV 71.0%	Negative	9	22
IL-33 + ADA + T-SPOT.TB	PPV 100.0%	Positive	50	0
	NPV 75.9%	Negative	7	22

The positive predictive value and negative value for IL-33, ADA, T-SPOT.TB were 100.0%, 43.1%; 96.4%, 83.3%; 89.8%, 80.0% respectively

*PPV* positive predictive value, *NPV* negative predictive value

## Discussion

Tuberculous pleurisy is the most common cause of pleural effusion, accounting for 49.5 to 54.5% of the cause of hospitalized pleural effusion in China [11]. A rapid and effective detection method for early diagnosis and treatment of patients with tuberculous pleural effusion is needed to reduce complications such as tuberculous empyema and lung damage caused by tuberculous pleural effusion [12].

IL-33 was discovered by Schmitz in 2005 and belongs to the IL-1 class cytokine superfamily. It has a homologous clover-like structures [13]. The results of this study showed that the level of IL-33 in pleural effusion was significantly higher in patients with tuberculous pleurisy than in non-tuberculous pleural effusion. The area under the ROC curve for IL-33 in TPE was 0.753, and the sensitivity was 49.1%, specificity is 100% at the best cutoff value of 155.96 ng/l. The level of pleural fluid IL-33 was positively correlated with pleural effusion ADA and peripheral blood T-SPOT.TB. Lee and Xuan and other scholars [9, 10] found that the level of IL-33 in pleural effusion of patients with tuberculous pleurisy was significantly higher than other causes of pleural effusion and serum IL-33 levels, the sensitivity was 78% and 86.96%, specificity was 65% and 90.48% respectively. It was also suggested by Lee and other scholars that pleural fluid IL-33 level and pleural ADA were significantly positively correlated [9]. Li and other scholars [8] also showed that the sensitivity of IL-33 in the diagnosis of tuberculous pleurisy was 83.9%, the specificity was 87.3%, and the area under the ROC curve was 0.823. Therefore, the above evidence shows that IL-33 is related to the pathophysiology of TPE. Although we did not probe the specific mechanism of IL-33 in the pathogenesis of TPE, the significant relationship between IL-33 and tuberculous pleurisy observed in this study can be explained by the following hypothesis: IL-33 is shown to exhibit an immunomodulatory effect to some extent, such as the induction of cytokines and responsive cells. A growing number of basic studies [14–16] have shown that IL-33 can mediate and even enhance Th1 cellular immune responses by increasing interferon-gamma (Interferon-γ, IFN-γ). On the other hand, some studies also found that IL-33 expression is up-regulated by IFN-γ and tumor necrosis factor-α (TNF-α) [17, 18]. IFN-γ is not only an upstream regulator of IL-33, but also a downstream product of IL-33 signaling [15, 16, 19]. Therefore, in tuberculous pleurisy, IL-33 and IFN-γ may form a coupled positive feedback loop [15–20]. Therefore, IL-33 may be involved in the pathogenesis and development of tuberculous pleurisy, and its elevated level may play a role in the stimulation of inflammation by mycobacterium tuberculosis. IL-33 has an important diagnostic value in the diagnosis of tuberculous pleurisy.

ADA is an important enzyme in the metabolism of purine nucleosides in various tissues of human body, especially lymphocytes. The pathogenesis of tuberculous pleurisy is delayed-type hypersensitivity caused by MTB infection. The tuberculosis protein of MTB enters the pleural cavity and causes pleural inflammatory reaction, which leads to lymphocyte differentiation and proliferation, resulting in increased ADA content [4]. ADA is one of the most widely studied and recommended biomarkers and has been found to have a good performance in diagnosing TPE. A meta-analysis of 63 studies [21] evaluated the value of pleural ADA activity in identifying TPE and non-TPE, demonstrating its high sensitivity and specificity (92% and 90%, respectively). ADA is one of the highly recommended biomarkers and has been found to have good performance in diagnosing TPE. The results of this study showed that the ADA level in pleural effusion was significantly higher in patients with tuberculous pleurisy than in non-tuberculous pleural effusion, the sensitivity of diagnosis of tuberculous pleurisy was 93.0%, the specificity was 90.9%, which was similar to the previous study [21]. Recently, some research showed some IgG4-related pleuritis cases with elevated adenosine deaminase in pleural effusion [22]. Combined diagnostic biomarkers can be useful in those specific situations.

Tuberculous pleurisy is mainly mediated by cellular immunity. After stimulation by MTB antigen, T cells get activated to secrete cytokine IFN-γ to participate in the immune response. There are corresponding specific T cells in the peripheral blood of patients [23]. The principle of T-SPOT.TB detection is to isolate MTB-specific T cells in peripheral blood, which can secret IFN-γ after in vitro culture and antigen re-stimulation, so we can diagnose the presence of MTB infection by examining the IFN-γ concentration with the corresponding antibody. Its diagnostic value is not affected by the patient’s sex, age, tumor, immunosuppression, etc. It can be used not only for the diagnosis of extrapulmonary tuberculosis, but also as a tool for therapeutic effect evaluation, which has a high practical value [24]. The results of this study showed that the level of T-SPOT.TB in peripheral blood of patients with tuberculous pleural effusion was significantly higher than that of patients with non-tuberculous pleural effusion. The sensitivity of peripheral blood T-SPOT.TB for diagnose of TPE was 92.3% and the specificity was 71.4%. However, studies have shown that peripheral blood T-SPOT.TB has its own defects because it is affected by the number of peripheral blood T lymphocytes [25], especially in immunodeficient patients [26], which may cause false negative results. Moreover, peripheral blood T-SPOT.TB cannot differentiate MTB latent infection from active tuberculosis, which further limits its use.

The results showed that the combined detection of these three indices, which required at least one of these measurements to be positive, resulted in an optimal sensitivity of 100%, whereas a specificity of 88.5% was found in a combination that required both of these three measurements to be positive. This study found that the combined detection of pleural effusion IL-33, ADA and peripheral blood T-SPOT.TB could further improve the sensitivity and specificity of TPE diagnosis. The area under the ROC curve was the largest at 0.962 when combined those three.

This study had several flaws, too. First of all, this study is a cross-sectional observational study; we didn’t follow up the patients and measure them again after TB treatment. Second, we did not analyze serial IL-33 levels or measure Th2 and Th1 cytokines such as IFN-γ in pleural effusion. Thirdly, we did not work on the detail mechanism. Further studies aiming to investigate the detailed mechanism can be carried out to confirm our findings in future.

In summary, this study compared the AUC, sensitivity and specificity of pleural effusion IL-33, ADA and blood TSPOT for the diagnosis of tuberculous pleurisy. We found that IL-33 has the highest specificity of 100%. It can be used for the exclusion of tuberculous pleurisy, but the sensitivity of IL-33 is not high, only at 49.1%. However, the diagnostic sensitivity was increased to 76.9% when combined with TSPOT.TB. Combined IL-33, ADA, TSPOT.TB, the diagnostic specificity was 100%, and the sensitivity was 88.5%, suggesting that the combined detection has a higher diagnostic value for tuberculous pleurisy. Gene xpert is widely used nowadays, but low sensitivity in pleural effusion limits its use in the diagnosis of tuberculous pleurisy. It is a cheap method to measure to IL-33, ADA, and blood TSPOT.TB with rapidity and convenience. It prevent missed diagnosis with so high specificity and sensitivity so we can treat patients without any delay. Those findings suggest that the combined test can be clinically utilized as an efficient diagnosis strategies for tuberculous pleurisy in clinics when we measure pleural effusion IL-33, ADA and peripheral blood T-SPOT.TB together.

## Conclusion

Combined detection of pleural effusion IL-33, ADA and peripheral blood T-SPOT.TB can improve the diagnostic efficacy of tuberculous pleurisy.

## Supplementary Information

**Additional file 1.** Raw data of patients, all characteristics of patients included.

## Data Availability

All data generated or analyzed during this study are included in this published article and its supplementary information file, Additional file 1.

## Abbreviations

IL-33: Interleukin- 33
ADA: Adenosine deaminase and
T-SPOT.TB.: Peripheral blood tuberculosis T cell spot detection
TB: Tuberculosis
IGRA: Interferon-γ release assay
TPE: Tuberculous pleural effusion
PPE: Pneumonia-like pleural effusion
MPE: Malignant pleural effusion
ELISA: Enzyme linked immunosorbent assay
LDH: Lactate dehydrogenase
ROC: Operating characteristic curve
SPSS: Statistical Product and Service Solutions
IQR: Interquartile range
MTB: Mycobacterium tuberculosis
